# ‘I'm Stronger and Healthier. Yeah, and More Fit’ Young Strong and Deadly Program Process Evaluation

**DOI:** 10.1002/hpja.70268

**Published:** 2026-10-29

**Authors:** Andrew Fraser, Tano D'Urso, Brett Biles, Patrick Kerrigan, LaWanna Warren, Rona Macniven, Stephanie Fletcher‐Lartey

**Affiliations:** ^1^ Faculty of Health University of Canberra Canberra Australia; ^2^ Integrated and Community Health, Western Sydney Local Health District Blacktown Australia; ^3^ Indigenous Division UNSW Sydney Kensington New South Wales Australia; ^4^ School of Population Health, Faculty of Medicine & Health UNSW Sydney Kensington New South Wales Australia; ^5^ Prevention Research Collaboration, Faculty of Medicine and Health The University of Sydney Camperdown Australia

**Keywords:** adolescent, Australian Aboriginal and Torres Strait Islander Peoples, exercise, family, health education, motivation

## Abstract

**Issue Addressed:**

Young strong and deadly is a health education and exercise program for Aboriginal young people aged 12–16 years in Western Sydney. The 14‐week program comprises paid gym memberships, guided gym sessions, transport provision, nutrition education and is led by Aboriginal qualified professionals. This mixed method process evaluation examined program delivery and implementation factors.

**Methods:**

Qualitative yarning participants were 19 program participants, six program staff and nine family member stakeholder participants post‐program and/or at 3‐ and 6‐months follow‐up. Quantitative data were collected through a program checklist tool that measured adherence and fidelity through observation and attendance, gym records and staff member characteristics.

**Results:**

In Cohort 1 (September–December 2023), 27 sessions were delivered over 14 weeks and an average of 90% (*n* = 19) of sessions were attended by 24/31 program completers. In Cohort 2 (May–August 2024), 26 sessions were delivered over 13 weeks and an average of 77% (*n* = 20) of sessions were attended by 13/18 program completers. Five themes reflect participant program acceptance and are described: Design and development of the Program; Program benefits and positive impact observed; Challenges and Barriers Experienced by the Program; Participant Motivations and Experiences; Future Directions and Recommendations for the Program.

**Conclusions:**

Young strong and deadly has demonstrated feasibility and acceptability among Aboriginal young people and their families.

**So What?:**

An assertive and culturally appropriate program that is purposeful and meaningful can be successfully implemented and improve the health and wellbeing of young Aboriginal people.

## 1 Introduction

Regular physical activity and a healthy diet during adolescence are key to preventing obesity and chronic disease that are experienced by Aboriginal and Torres Strait Islander people at higher rates and at a younger age than non‐Indigenous people [1]. While Aboriginal and Torres Strait Islander people have had healthy diets and physically active lifestyles for many thousands of years, the disruption of food systems and ongoing negative effects of colonisation have led to unhealthy diets of young people today [2, 3]. Obesity is related to social determinants of health; a healthy body composition was less common among adolescents experiencing overcrowded housing and receiving income support [4]. Higher levels of physical activity are associated with a healthy body composition [5]. However, the high physical activity levels decline during adolescence [6, 7] while the prevalence of overweight and obesity increases [8].

Social and emotional wellbeing (SEWB), holistic cultural connection to body, mind and emotions, family and kinship, community, culture, Country and spirituality and ancestors [9] is positively associated with young people's physical activity [10]. Physical activity programs for Aboriginal and Torres Strait Islander young people can increase connection to culture, self‐esteem and confidence [11]. Aboriginal leadership in the design and implementation of programs to improve child and adolescent physical activity and nutrition and prevent obesity is vital [2, 12].

### 1.1 Young Strong and Deadly Program

The Young Strong and Deadly program is a 14‐week voluntary health education program focusing on strength exercise and nutrition for Aboriginal and Torres Strait Islander people aged 12–16 years old in western Sydney (Appendix 1). Co‐designed with Western Sydney Aboriginal communities and led and delivered by qualified Aboriginal staff to ensure cultural authority, centrality and governance, the program's purpose is to reduce modifiable disease risk factors for young Aboriginal people by supporting participants to establish routine physical activity, improve diet and social and emotional wellbeing. Twice per week, participants were guided and supported through validated muscular strength building exercises. Food and nutrition literacy sessions included facilitated yarning circle discussion [13, 14] (with accompanying informative PowerPoint slide presentations) and information sharing on macronutrients' impact on health and basic nutrient exchange practices. To solidify these learnings, cooking activities with participants and families were conducted, utilising facilities and toolkits from the National Heart Foundation's Koori Cook‐Off program. Further detail is found in the Program Logic, which includes description of inputs, activities, outputs and outcomes (Appendix 1). Cohort 1 was delivered between September and December 2023 and Cohort 2 was delivered between May and August 2024 (Appendix 1). An impact evaluation assessed pre‐post changes in physical health, activity and nutrition behaviours and program acceptability [15]. Preliminary results show the program was well received by participants and achieved its intended short‐term objectives as demonstrated by significant improvements in strength and conditioning, diet quality, behaviours, food and nutrition literacy, social and emotional wellbeing measures [16].

### 1.2 Process Evaluation

This process evaluation study aimed to examine program delivery and implementation factors of the Young Strong and Deadly program. Process evaluation allows for an in‐depth exploration of program delivery and how the program context, including community priorities, program beneficiaries and the mechanisms or pathways through which the program may affect outcomes [17]. The inclusion of process evaluations in studies with Aboriginal participants can give an understanding of program delivery and implementation factors in Aboriginal community contexts [18].

## 2 Methods

The first two cohorts in the program were implemented by the Western Sydney Local Health District (WSLHD) in collaboration with relevant community organisations within the WSLHD catchment. A combination of convenience and snowball sampling approach was used for recruitment. Electronic and paper flyers containing YSDP information, eligibility criterion and registration instructions were distributed to schools, malls and relevant organisations within the Blacktown and Penrith Local Government area. Distribution included encouragement to share with relevant networks.

### 2.1 Design and Approach

The process evaluation was guided by Aboriginal ways of knowing, being and doing [19], which acknowledges the historical, present and ongoing impact of Western colonisation on Aboriginal peoples. This process evaluation includes privileging Aboriginal perspectives, experiences and knowledge systems whilst challenging and resisting dominant epistemologies.

A mixed methods approach was used [15] comprising a process (implementation) evaluation, guided by the Program Logic (Appendix 1).

Yarning is a well‐known relaxed form of communication/storytelling with Aboriginal people and is used in everyday interactions with other Aboriginal people; it is the way we make sense of our lived experience [14]. There are four types of yarning: social yarning; research topic yarning; collaborative yarning and therapeutic yarning [15]. Social yarning (conversation that takes place before the research or topic yarn) and research topic yarning (relaxed but purposeful, to gather information related to the research topic) were included to [13] help facilitate in‐depth discussions that result in thick descriptions. While this may also be common with other qualitative data collection methods, yarning offers Aboriginal people a more relaxed approach whereby they can talk freely about their experiences [14]. The yarning sessions were facilitated by three Aboriginal research assistants and one Aboriginal and one non‐Aboriginal researcher with physiotherapy, physical education, sport science and post‐doctoral (PhD) qualifications.

### 2.2 Participants

Thirty one participants were recruited to Cohort 1; 24 of whom (8 girls and 16 boys; mean age 14.0 years) completed the 14 week program [16]. Eighteen participants were recruited to Cohort 2 (4 girls and 14 boys; mean age 16.2 years), seven of whom had also participated in Cohort 1. Thirteen participants completed Cohort 2. Eligible participants for this process evaluation were program participants from Cohort 1 and Cohort 2. Stakeholder participants were also recruited through purposeful sampling [20] of six program delivery and managerial staff from WSLHD and Mount Druitt PCYC and nine family stakeholders during both Cohorts.

In December 2023, 19 of the 24 (79%) Cohort 1 program completers (5 girls and 14 boys) took part in five different yarning groups, with 2–6 young people in each group. Eight parent/carers (all women; participants' mothers and aunties) also took part in post‐program yarns, two individually and three in pairs. Of the program participants who took part in the post‐program yarning, 10 (3 girls and 7 boys, six of whom participated in both cohorts) also took part in the 3‐month follow‐up yarning in March 2024, one group with three participants, one group with five participants and a third group with two participants and their parent/carer, due to family preferences. In addition, two stakeholders involved in program delivery also participated in individual yarns. In September 2024, five program stakeholders participated in an individual yarn, one of whom had also participated in a 3‐month follow‐up yarn.

### 2.3 Data Collection

Data were collected by three Aboriginal research assistants and one Aboriginal and one non‐Aboriginal researcher with physiotherapy, physical education, sport science and post‐doctoral (PhD) qualifications and experience in conducting evaluation studies with Aboriginal people, health services, not‐for‐profit and community‐controlled organisations.

At the end of week 12 of Cohort 1, a program checklist tool [18] adapted by the researchers and program staff was applied by the researchers (one Aboriginal research assistant, one Aboriginal and one non‐Aboriginal post‐doctoral researchers) to measure program adherence and fidelity through direct observation of a program session. This tool assessed whether sessions were consistent with the program components and to assess the quality of delivery, including the concepts, content, activities, engagement and delivery strategies.

Participants for the qualitative yarning data collection, facilitated by each researcher, at the end of the 14‐week program delivery period (Cohort 1 post‐test) were contacted by the researchers by phone during the final week of the program and invited to take part in a semi‐structured yarning focus group. Individual or group yarns with family member stakeholders who attended the facility to collect their children participating in the program were also conducted to determine program context and potential sustainability. Yarning questions explored barriers and enablers, and perceived changes that occurred since the start of the program as well as views and experiences of the program and its perceived effects (Appendix 2). At 3‐month follow‐up (March 2024) after the end of the 14‐week program delivery, Cohort 1 participants and family stakeholders were contacted by the researchers by phone and invited to take part in the follow‐up yarns. Yarning interviews took place at the PCYC in closed rooms or halls, food was provided for participants and transport was provided for where required.

At the end of week 12 of Cohort 2 (August 2024), the adapted program checklist tool [18] was again applied by the researchers to measure program adherence through direct observation of a program session. At the end of Cohort 2, program stakeholders were also invited to take part by phone and email in yarning interviews at the PCYC or online through Microsoft Teams, depending on their preference. Yarning questions explored stakeholder experiences of the program, their perceptions of its impact on participants and attendance challenges, program appropriateness and implementation challenges (Appendix 2).

## 3 Data Analysis

A decolonising lens was applied to the analysis through privileging Aboriginal voices, knowledge and practices [19, 21]. Cohort 1 program attendance records were reviewed for all enrolled participants to determine participant reach and maintenance of involvement in the 14‐week program. PCYC gym attendance records were reviewed for Cohort 1 participant attendance between January and September 2024. The characteristics and qualifications of staff members involved in Cohort 1 and Cohort 2 program delivery were also documented.

Qualitative group yarns were transcribed by a professional data transcription service and Microsoft Teams. Transcripts were reviewed and analysed by the researchers using thematic analysis. Quantitative results were analysed using descriptive statistics. The results were interpreted to gain a multilayered understanding of the facilitators of, and barriers to, program uptake and of program acceptability and to provide future recommendations.

Ethics approval was obtained from the Sydney Children's Hospitals Network (SCHN) Human Research Ethics Committee (2023/ETH00583) and the Aboriginal Health & Medical Research Council Human Research Ethics Committee (2124/23). Informed consent was obtained from all participants.

## 4 Results

### 4.1 Participant Attendance

In Cohort 1, 27 sessions were delivered over a 14‐week period (Table 1). The mean number of sessions attended by the 24 participants who completed the program was 19/27 sessions delivered (70%). The mean number of participants attending each session was 16/24 participants (67%), noting that two participants did not commence the program until Session 9. In Cohort 2, 26 sessions were delivered over a 13‐week period. The mean number of sessions attended by the 13 participants who completed the program was 20/26 sessions delivered (77%). The mean number of participants attending each session was 10/13 participants (77%).

**TABLE 1:** Program attendance.

Cohort	Number of participants		
	Lower attendance (0–9 sessions)	Middle attendance (10–18 sessions)	Higher attendance (19–27 sessions)
1	1	9	14
2	0	4	9

PCYC gym attendance records from the immediate post‐cohort period of January–September 2024 when participants had received their 6‐month free memberships indicated that 8/24 (33%) of Cohort 1 participants had attended the gym post‐program and that the 8 participants had attended between 2 and 19 times (mean 7 times; Table 2).

**TABLE 2:** Cohort 1 post‐program PCYC gym attendance (January–September 2024).

Number of participants	Number of PCYC attendances
1	19
1	10
1	8
1	7
1	5
2	4
1	3
1	2
Total: 9	Total: 58

### 4.2 Staff Member Characteristics and Qualifications

In Cohort 1, four staff members were involved in delivering the program, including supervising gym and nutrition education sessions, providing instruction, liaising with schools and families and transporting participants to and from the program. Three were Aboriginal, two of whom were Australian Strength and Conditioning Association (ASCA) Level 1 Coaches, and one of whom was also a qualified Aboriginal Health Practitioner, held a Bachelor of Food and Nutrition Science degree and ASCA Level 1 accreditation. Three staff members delivered Cohort 2, two who assisted in the delivery of Cohort 1 took a leadership role in Cohort 2 (one Aboriginal, one non‐Aboriginal), both of whom were working towards the ASCA Level 1 Coaches qualification at the time of Cohort 2.

### 4.3 Program Adherence: Cohort 1 and 2

The results of the Cohort 1 and Cohort 2 program checklist tool are presented in Table 3. While the checklists provide quantitative scores, they should be considered within the context of the qualitative observations. In both cohorts, it was evident from the quantitative scores that most program elements were delivered in the session observed, with 19/26 (73%) elements scoring ‘yes’ in Cohort 1 and 16/26 (62%) elements scoring ‘yes’ in Cohort 2. In both cohorts, two nutrition‐focused sessions were not observed due to a lack of researcher availability to attend these sessions but had been delivered in recent weeks. It was apparent that staff were working well together in both cohorts and relating well to participants. Mutual respect was evident.

**TABLE 3:** Program checklist tool results.

Cohort 1		
Key element	Completed (Y, N, N/A)	Comments
The session activities included		Coach did a general unstructured yarn (check in/welcome) with participants? Yes, informally as participants arrived Describe the mood of the room? (calm, excited, busy, tense, is there nervous laughter, is there awkward silence?) Motivated and calm Do participants appear happy and comfortable? Yes very Listen to what is being said. Note language/words being used Participants respectfully engage with each other and staff. Language is positive and supportive Note the ways in which participants and coaches interact? Respectfully, listening to each other even in a noisy environment How do participants interact with each other? Respectfully and in an organised way in small groups Is the facility/equipment suitable? Yes, a range of equipment for different exercises and ability. All safe and in good working order Are there additional Aboriginal cultural components that we haven't captured elsewhere? Nothing additional
Rules, responsibilities and program expectations explained to participants	Unclear	
Warm up and cool down	Y	
Coach observes and critiques participant exercise technique	Y	
Coach provides explanation, guidance and feedback	Y	
Coach provides informal information on anatomy and physiology	Y	
Coach provides positive reinforcement	Y	
Instruction on gym equipment	Y	
Emphasis on opportunities to participate in regular physical activity	N	
Learning about relevant health services	N	
Emphasis on challenging yourself (no set number, time or intensity)	Y	
Emphasis on being safe	Y	
Coaches provide relevancy of exercises to everyday adaptations i.e., sports	Y	
Recap of how to improve strength e.g., increase reps, do different exercises, use weights/bands	Y	
Recap of how to improve fitness e.g., increase frequency, duration, intensity	Y	
Coach demonstrates new and existing exercises	Y	
Participants performs new and existing exercises	Y	
Participants provide feedback on muscle activation	Y	
Learning about healthy eating	Not this week but previously	
Providing resources for healthy eating		
The opportunity to practice food preparation and cooking		
Overall		
Participants are engaged in exercise component	Y	
Participants are engaged in the nutrition component	N/A	
Participants ask coach questions	Y	
Staff appear to be working well together	Y	
Session facilitated in a culturally appropriate way	Y	
Coach respectful of all participants	Y	
Additional comments you would like to tell us about the session today/any suggestions you would like to make: Rules, responsibilities and program expectations were not explicitly explained to participants, but participants seem clear on these aspects by week 10 Kids are mainly performing free weights, with some machine weights also and exercises like planks in the next door boxing area, plus shuttle sprints outside Detailed records were kept by staff on participants' previous lifting weight levels Kids are organised into groups roughly by age and also gender Police staff member is also working directly with kids Kids are choosing their own music to play in the background While the observed session did not include a nutrition component, three sessions of cooking education and skills recently took place at the PCYC and Kimberwalli (local community controlled service) as confirmed by participants and program staff		

Cohort 2		
Key element	Completed (Y, N, N/A)	Comments
The session activities included		Coach did a general unstructured yarn (check in/welcome) with participants? Yes, 1–1 or in small groups. Session began with 20min informal basketball and chats with staff, supporting any pressing, broader concerns e.g., participant applying for a driving test Describe the mood of the room? (calm, excited, busy, tense, is there nervous laughter, is there awkward silence?) Teenagers are focused and happy to be there Do participants appear happy and comfortable? Yes, overall a good vibe Listen to what is being said. Note language/words being used Participants respectfully engage with each other and staff. Language is positive and supportive Note the ways in which participants and coaches interact? It is clear there are good relationships, two‐way respect How do participants interact with each other? Also respectfully and positively, there are some clear friendships. Kids encourage each other Is the facility/equipment suitable? Yes, a range of equipment for different exercises and ability. All safe and in good working order Are there additional Aboriginal cultural components that we haven't captured elsewhere? Nothing additional
Rules, responsibilities and program expectations explained to participants	N	
Warm up and cool down	Y	
Coach observes and critiques participant exercise technique	Y	
Coach provides explanation, guidance and feedback	Y	
Coach provides informal information on anatomy and physiology	Unclear	
Coach provides positive reinforcement	Y	
Instruction on gym equipment	Y	
Emphasis on opportunities to participate in regular physical activity	Unclear	
Learning about relevant health services	N	
Emphasis on challenging yourself (no set number, time or intensity)	Y	
Emphasis on being safe	Y	
Coaches provide relevancy of exercises to everyday adaptations i.e., sports	Unclear	
Recap of how to improve strength e.g., increase reps, do different exercises, use weights/bands	Y	
Recap of how to improve fitness e.g., increase frequency, duration, intensity	Y	
Coach demonstrates new and existing exercises	Y	
Participants performs new and existing exercises	Y	
Participants provide feedback on muscle activation	N	
Learning about healthy eating	Not this week but previously	
Providing resources for healthy eating		
The opportunity to practice food preparation and cooking		
Overall		
Participants are engaged in exercise component	Y	
Participants are engaged in the nutrition component	N/A	
Participants ask coach questions	Y	
Staff appear to be working well together	Y	
Session facilitated in a culturally appropriate way	Y	
Coach respectful of all participants	Y	
Additional comments you would like to tell us about the session today/any suggestions you would like to make: Rules, responsibilities and program expectations were not explicitly explained to participants, but participants seemed clear on these aspects by week 10 Learning about relevant health services was not apparent in this session but other services were discussed e.g., job services Where ‘unclear’ is selected, the observer did not specifically observe these elements occurring but they may have occurred in specific conversations beyond the earshot of observer in a different parts of the gym or outside The session commenced with informal basketball in the PCYC hall between 2 and 2:20 pm followed by an outdoor lap of the building as a warm up 20 kids were recruited to the cohort, 1/3 new, 10 attending today, 20 started, 1 had to drop out due to working 30 h weeks after parents became unemployed There were approx. 5 other teenagers in gym doing independent unstructured exercise Police staff member was working directly with kids for a few minutes at the start of the session Exercises performed: Deadlift, leg press, barbell (legs focus today (Tuesday), arms on Thursday). Shuttle runs done on basketball court (named ‘suicide’) Compared to Cohort 1 observation, staff had less supporting paperwork like checking participants' previous lifting weight level 2 sessions focussed on cooking, and vaping education had recently been completed Kids were helping with program activities e.g., changing weight levels There were posters in PCYC for their Wednesday morning 7 am fitness and breakfast program Staff mentioned exercise height and weight being measured pre‐post program but not research surveys as staff were unaware this was to happen beyond Cohort 1 Staff said 10/15 other kids were interested in taking part		

### 4.4 Qualitative Yarning Interviews

Five themes with subthemes were identified in the analysis of the participant and family post‐program and 3‐month follow‐up, and program stakeholder yarning interviews. Quotes are identified as participants, parent/carers with the relevant timepoint (post Cohort 1, Cohort 1, 3‐month follow‐up) or program stakeholders. Figure 1 gives a visual depiction of the qualitative yarning interview themes and subthemes and the connections between themes. The program benefits and strengths were linked to the co‐design and localised development of the program. The program was co‐designed with Western Sydney Aboriginal communities and led and delivered by qualified Aboriginal staff to ensure cultural authority, centrality and governance. Given that participants were teenagers, their contributions within the yarning circles were often brief and direct, reflecting communication styles typical of this age group, where meaningful perspectives may be expressed through concise rather than extended verbal responses.

**FIGURE 1:**
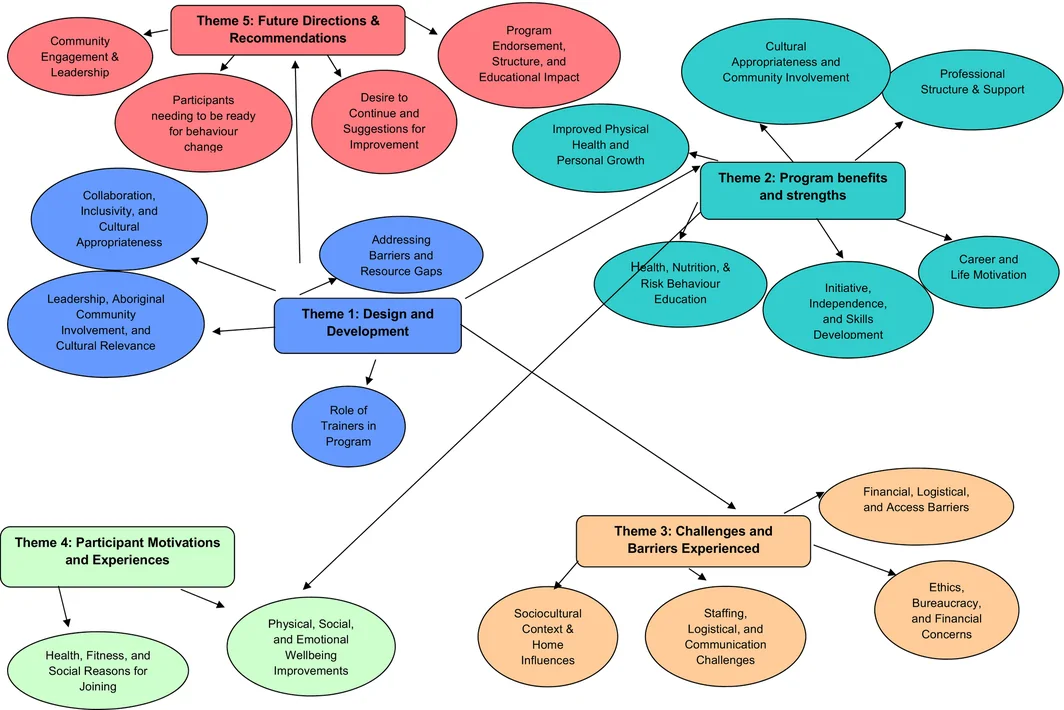
Young strong and deadly program qualitative yarning themes.

### 4.5 Theme 1: Design and Development of the YSD Program

Program design addressed physical activity and program barriers and resource gaps that had been previously identified in formative and pilot development such as transport and costs to participants through collaborative, inclusive and culturally appropriate strategies.

#### 4.5.1 Subtheme: Addressing Barriers and Resource Gaps

Both practical and structural barriers to young people's participation in physical activity were described. Transport was important for participants who relied on others to attend. The provision of transport was therefore viewed as an access facilitator, with the PCYC community bus described as ‘so much help’ (participant, post program) because ‘some of the people don't have lifts or anything’ (participant, 3 month follow up).

Stakeholders reflected on identifying and responding to broader challenges, recognising ‘barriers to physical activity for kids’ and ‘identifying gaps in the existing literature that needed to be addressed’ (program stakeholder). Financial accessibility was another consideration. Stakeholders described ‘advocating assertively for resources and funding’ and seeking to *‘*eliminate (the) financial barrier for (the) first six months after (the) program*’* (program stakeholder).

#### 4.5.2 Subtheme: Collaboration, Inclusivity and Cultural Appropriateness

Program participant highlighted the importance of an inclusive and culturally comfortable environment. Being alongside other Aboriginal young people was particularly valued, with one noting that ‘we're surrounded by people (who) are like, our culture’ (participant, post program). Another described the program as ‘always inclusive and welcoming’ (participant, post program). These comments suggest that the social inclusiveness of the program and the contribution of culture to a sense of belonging were valued.

Stakeholder reflections indicated that cultural appropriateness was also considered during program development and refinement through ‘consultation with researchers and academics’ and adapting program materials in response to participants' engagement. For example, ‘poor responses to an initial questionnaire (resulted in it being) edited to be more culturally appropriate’ (program stakeholder). This demonstrates an iterative approach in which program delivery and evaluation were modified in response to the needs and experiences of Aboriginal young people.

#### 4.5.3 Subtheme: Leadership, Aboriginal Community Involvement and Cultural Relevance

Stakeholders identified Aboriginal leadership, experience and local contextual understanding as important to program delivery. Trainers were described as having a ‘passion for working with young people, especially in sport and fitness’, and experience working ‘with young people in disadvantaged contexts’. Their role extended beyond providing exercise instruction, to ‘providing kids with a role model and guidance’ (program stakeholder). The capacity of trainers to deliver activities ‘in a culturally safe way’ was also identified as important and that ‘there was quite a bit of commitment and this was Aboriginal leadership at its best’.

Stakeholders also identified limitations in Aboriginal community involvement in some implementation aspects. While the program was described as responding to an ‘urgent need in community’ (program stakeholder), concerns were raised about Aboriginal leadership and consultation in the second cohort withone stakeholder identified ‘no community involvement or consultation’.

#### 4.5.4 Subtheme: Role of Trainers in Program Success

The young people indicated that trainers were vital in developing their knowledge, confidence and capacity for physical activity participation. They described entering the program with limited gym‐based exercise knowledge and developing understanding through participation. As one explained, ‘When we first started, we didn't know much about gym and now we do’ (participant, 3 month follow up). Another reported that trainers ‘taught us new things I didn't know’ (participant, post program), indicating that the program provided opportunities to develop practical knowledge that could support continued physical activity.

Participants also valued the practical instruction provided by trainers, including ‘helping with form and guidance’ (participant, post program), suggesting that trainers supported the development of appropriate exercise techniques rather than simply supervising. Trainers were also viewed as a source of motivation, with one participant explaining that ‘they push you to go as hard as you can as well’ (participant, post program) and a subsequent reflection that ‘it is harder to do it by yourself’ (participant, 3 month follow up). Together, these accounts suggest that trainers contributed to program success through a combination of technical instruction, encouragement, motivation and interpersonal support.

### 4.6 Theme 2: Program Benefits and Positive Impact Observed

Program participants, parent/carers and stakeholders described a range of positive changes associated with program participation. These extended beyond improvements in physical activity and fitness to include confidence, emotional wellbeing, diet, independence, social connection and practical skill development. Culturally appropriate delivery, relationships with trainers and the structured nature of the program contributed to participant engagement and experiences.

Participants and parent/carers described changes in physical activity beyond the program environment to everyday life. For some young people, participation appeared to encourage new physical activities. One participant explained, ‘Yeah I go ride my bike… I never used to do that. I do that now’ (participant, post program). There was also evidence that physical activity continued after the formal program had ended, with another stating, ‘Yeah I continue to do exercise… I just don't use the machines or anything’ (participant, 3 month follow up).

The reported benefits also extended to emotional wellbeing and confidence with exercise as something that ‘makes me feel better. Like in myself’, contributing to ‘feeling happy with myself’ (participant, post program). Parent/carers observed similar changes, including improved confidence, assertiveness and school engagement, with one describing ‘increased confidence with substantially improved school attendance’, while another that their child was ‘sticking up for herself a lot more now… a lot more assertive’ (parent/carer, post program). Stakeholders also reported ‘increased emotional wellbeing’, ‘huge improvement in confidence’.

#### 4.6.1 Subtheme: Cultural Appropriateness and Community Involvement

Cultural relevance was identified as an important with stakeholders emphasising the value of having trainers who could understand and relate to the program participants, described as ‘Indigenous men who understood the children’ (program stakeholder). This suggests that cultural connection was embedded both through program content and delivery staff, embodied by relationships developed with participants.

Cultural connection was also incorporated through practical activities. Stakeholders highlighted opportunities for participants to ‘learn to cook culturally using cultural ingredients’, while older young people were observed ‘taking on mentoring roles’. These elements indicate that the program provided opportunities for cultural learning and peer support alongside its health and fitness components. The involvement of Aboriginal trainers and opportunities for older participants to act as mentors also demonstrate how cultural and community relationships contributed to the broader program environment.

#### 4.6.2 Subtheme: Initiative, Independence and Skills Development

The program also appeared to support participants to develop greater independence, motivation and practical skills. Participants described becoming more proactive in relation to physical activity and healthy eating. One young person characterised this change as ‘just getting up and doing it,’ contrasting this with being less active before the program, and described helping to prepare ‘healthier food and better foods’ (participant, post program). These reflections suggest that participants were beginning to apply some of the knowledge and behaviours developed through the program independently.

Stakeholders similarly observed young people taking greater initiative in relation to their health and fitness. At 3 month follow up, one stakeholder described participants being ‘pretty motivated to engage with PCYC fitness activities independently, ask questions and seek advice from personal training staff’. This was viewed positively as evidence of continued engagement and collaboration beyond the immediate program. Stakeholders also identified ‘helping build independence’ and ‘building community networks (outside the program)’ as important outcomes. Together, these findings suggest that the program supported participants not simply to follow prescribed activities, but to progressively develop the confidence and skills to make decisions and seek support independently.

Stakeholders also observed changes in participant interaction during the program. These included ‘improved cooperation and communication with other kids’ and more ‘active participation in group discussions’ (program stakeholders). These observations indicate that the group‐based program provided opportunities for young people to practise communication, cooperation and participation in a social environment.

#### 4.6.3 Subtheme: Professional Structure and Support

The structured program nature and professional support provided were also identified as strengths. Participants valued trainers ‘explaining what we needed to do’ (participant, post program), to support their engagement. Stakeholders similarly highlighted that the program was ‘trained and designed by a professional’ and participants knew ‘what to expect each day’ (program stakeholders).

#### 4.6.4 Subtheme: Health, Nutrition and Risk Behaviour Education

Participants described noticeable changes in their and dietary knowledge and behaviours, with one explaining, ‘I don't eat as much cookies… I've been cutting out on a lot of food… I'll buy some fruits’ (participant, post program). Another compared their family's previous regular fast food purchasing with preparing healthier meals at home: ‘Before the program I remember we used to get Maccas every day… Now after the program we just make healthy foods at home’.

One parent/carer also observed that their child had become ‘more conscious of her intake and what she actually puts in her mouth’ (parent/carer, post program), while a participant reported at 3 month follow up that their family ate ‘fewer lollies’ and that the program had changed ‘our routine and like our diet.’ These accounts suggest that nutrition education may have influenced not only individual knowledge but family and home behaviours.

Stakeholders described ‘introducing young people to new foods and concepts’, alongside practical skills such as ‘teaching kitchen safety’. In Cohort 2, health education extended beyond nutrition to ‘vaping and other risky behaviours’. ‘Family and community involvement in nutrition sessions’ further broadened the reach of this component. Together, the findings indicate that health education was integrated into the program as a practical component designed to equip young people with knowledge and skills that could be applied beyond the program setting.

#### 4.6.5 Subtheme: Improved Physical Health and Personal Growth

Participants, parent/carers and stakeholders described physical fitness improvement alongside broader personal growth like ‘building up strength, building our bodies’ and social benefits such as ‘making new friends’ (participant, post program). One example of behavioural change was reported at 3 month follow up by a participant who previously relied on family for transport to school despite living within a short walking distance, reporting ‘ever since the program I have walked to school nearly every day to and from’. This suggests that increased physical activity may have become incorporated into everyday routines rather than remaining limited to formal exercise sessions.

Parent/carers also observed increased motivation to continue physical activity after the program, including young people wanting to ‘attend and use their gym memberships’. Stakeholders similarly identified improvements in ‘strength, speed, fitness (and) wellbeing,’ alongside greater willingness to ‘push themselves and try new activities’.

Personal growth was also reflected in increased confidence and willingness to move beyond comfort zones. Stakeholders described participants as becoming ‘more comfortable and confident in themselves’ and motivated to pursue opportunities such as ‘employment and independent exercise’.

### 4.7 Theme 3: Challenges and Barriers Experienced by the Program

Despite the positive outcomes reported, program implementation was affected by operational and structural challenges. These included staffing and communication issues, administrative and financial requirements, participants' broader home and social circumstances and practical barriers to program accessing that influenced outcomes.

#### 4.7.1 Subtheme: Staffing, Logistical and Communication Challenges

Stakeholders identified staffing capacity and communication as program implementation challenges. At times, limited staffing placed considerable demands on those involved in delivery, with one stakeholder describing staff as ‘going without breaks, working around the clock’ (program stakeholder), suggesting that the resources required for program implementation were sometimes greater than what was provided.

Staff communication was also identified as an area requiring improvement. Stakeholders described in ‘staff‐staff communication’ challenges as well as between ‘previous, new and current’ staff members.

#### 4.7.2 Subtheme: Ethics, Bureaucracy and Financial Concerns

Stakeholders identified structural and administrative requirements as challenges to establishing and maintaining the program. The research ethics process was described as ‘difficult’, alongside the broader ‘bureaucracy of forming a program and conducting research’ (program stakeholder). Financial constraints represented a further challenge. Stakeholders referred to the ‘financial barriers of conducting a program’ and raised concerns about ‘sustainability, particularly when existing transport fees are removed’.

#### 4.7.3 Subtheme: Sociocultural Context and Home Influences

Stakeholders recognised that young people's capacity to participate in and benefit from the program was influenced by external circumstances. This included ‘trauma and stuff happening at home’, competing commitments with ‘some kids participating in other athletic programs’. One emphasised the importance of the broader family environment and how engagement was shaped by family circumstances, social relationships and the broader environments in which young people lived., stating that ‘the house (home life) has to be in order first for the program to be implemented and successful’ (program stakeholder).

#### 4.7.4 Subtheme: Financial, Logistical and Access Barriers

Practical access to the program was another important challenge. The value of program transport provision was quite significant. Stakeholders identified ‘infrastructure and resource’ limitations, as well as ‘minimum age requirements for gym use’, as factors affecting participation. The impact of transport barriers was clearly illustrated by one participant who explained, ‘I have to walk like an hour. I can't really get driven anywhere because someone has to look after my sister at home, my baby sister’ (participant, 3‐month follow‐up). Another participant described how ‘some of the people don't have lifts or anything’ (participant, 3 month follow up). Transport barriers intersected with participants' family circumstances and directly affect program access.

### 4.8 Theme 4: Participant Motivations and Experiences

Participants described joining the program for a combination of health, fitness and social reasons. Their reflections indicate that participation was associated with perceived improvements in physical and emotional wellbeing, behaviours and confidence. Importantly, some participants described changes that continued after the scheduled program ended, suggesting a longer‐term influence on attitudes and behaviours.

#### 4.8.1 Subtheme: Health, Fitness and Social Reasons for Joining

Improving health and fitness was an important initial motivation. For some participants, the program provided an opportunity to become more active despite limited previous involvement in structured exercise, with one participant described joining because they wanted to ‘be a little bit more healthier’ (participant, post program).

For others, participation was driven by curiosity and a willingness to try something new. One participant explained, ‘I didn't do that much like fitness. But when I heard of this, I thought why not try it out. And I loved it while I was doing it’ (participant, post program). This shows a progression from limited previous engagement in fitness and initial willingness to participate to enjoyment of the program.

Stakeholders also observed that the program offered some young people opportunities to develop independence and take greater ownership of physical activity. One stakeholder described older participants? embracing the ‘freedom’ associated with coming to the gym, exercising and making their own way home, observing that ‘you see those kids grow up very quickly’ (program stakeholder).

#### 4.8.2 Subtheme: Physical, Social and Emotional Wellbeing Improvements

Participants reported perceived improvements in both physical and emotional wellbeing in an interconnected way following participation. Physical changes were described in terms of increased strength, fitness and general health. One participant explained, ‘I'm also stronger and healthier. Yeah, and more fit’ (participant, post program), while another stated that the program had ‘improved my fitness (both) physically and mentally’ (participant, post program).

There was also evidence of sustained behavioural changes. At 3 month follow up, one participant reflected on changes in their eating behaviour, explaining that they used to ‘binge eat a lot……but now I don't eat as much but I still do eat a lot of good nutritional stuff’. Another participant reported ‘Confidence big time’ when reflecting on changes 3 months after the program.

### 4.9 Theme 5: Future Directions and Recommendations for the Program

Participants, families and stakeholders identified several considerations for future program development and sustainability. While stakeholders expressed support for the program and its structured approach, findings also highlight the importance of community leadership, continued access to physical activity opportunities and practical support for participants and families.

#### 4.9.1 Subtheme: Program Endorsement, Structure and Educational Impact

Stakeholders expressed support for the program and perceived it as producing meaningful changes for participants. One stakeholder stated that they ‘would recommend this program,’ while another identified its ‘proven ability to make a significant impact on individual level’ (program stakeholders).

The structured program nature was identified as important to perceived impact. Stakeholders highlighted the inclusion of a period of ‘health education’ alongside a planned exercise program, including ‘set exercises during the first four weeks and specific areas targeted on different days’ beyond just an opportunity for young people to attend a gym.

#### 4.9.2 Subtheme: Community Engagement and Leadership

Stakeholders identified stronger community involvement and leadership as important considerations for future program delivery. One explicitly stated that ‘community run and involvement is better than government in this case’, indicating a preference for greater community ownership of program implementation.

This was accompanied by recognition of the ‘need for capacity building’ within the community, emphasising how local capacity can be strengthened to support greater involvement in leadership and delivery. This is particularly relevant to the concerns identified elsewhere in the findings regarding periods of implementation that were perceived as having insufficient Aboriginal community involvement.

#### 4.9.3 Subtheme: Participant/Family Desire to Continue and Suggestions for Improvement

Participants expressed an interest in continuing physical activity after the formal program, but also identified practical barriers to continued participation. Financial accessibility was particularly important. One participant explained, ‘I like training… but also some of us don't have enough money to buy memberships’. This suggests that motivation to continue exercising did not necessarily translate into the ability to maintain gym access beyond the program.

Participants also highlighted the social dimension of continued participation with one explaining that ‘it would be kind of weird going to the gym without people’ (participant, post program).

Parent/carers similarly identified logistical challenges. One explained that ‘to get him here to the program is hard for me, especially if I'm in the office that day’, demonstrating how participation could depend on family availability and transport arrangements. This reinforces findings elsewhere in the study regarding the importance of transport support.

Young people also offered practical suggestions for improving or extending the program. These included introducing ‘new recipes… recipes we can give to our parents,’ providing ‘longer gym memberships and access to appropriate gym shoes’. These suggestions are important because they demonstrate participants identifying specific resources that could help transfer program learning into their home lives.

There was also evidence that some participants continued exercising even without access to all the resources available during the program with one participant reporting ‘Yeah I continue to do exercise… I just don't use the machines or anything’ (participant, 3 month follow‐up). This suggests that some knowledge or motivation developed through the program was maintained, while also highlighting how access to equipment and facilities may influence the type of physical activity participants can continue.

## 5 Discussion

This mixed method process evaluation provides evidence that Young Strong and Deadly is a feasible and acceptable program for Aboriginal young people and their families, with additional evidence of stakeholder support for the program. Some challenges were experienced by participants, families and stakeholders that impacted program continuity and sustainability; however, the identification of these issues allows for collaborative solution generation. Process evaluation of programs for Aboriginal young people is rare and this study offers vital insights to guide future program design and implementation for physical activity and nutrition programs as well as broader health and wellbeing programs. This type of evaluation gives a more comprehensive understanding of the different parts of the process evaluation as well as the implementation factors associated with program efficacy in improving strength and conditioning outcomes [16].

Attendance data demonstrated high engagement through the program delivery period. Qualitative yarning results suggest that this is likely due to the welcoming, culturally safe, fun and engaging program led by qualified staff who could relate to, and inspire, Aboriginal young people as well as the social, group program aspects. Additional assertive strategies to address known barriers such as transport and finance facilitated retention across both cohorts. These findings are consistent with a process evaluation of a group exercise and nutrition education program for Aboriginal and Torres Strait Islander women who found the program met their needs and expectations and there was a positive class atmosphere where they developed relationships with other participants and program staff [22].

Program checklist findings indicated high program fidelity and implementation in delivering a culturally appropriate, guided physical activity program that taught young people how to perform physical activity safely and effectively in a sport and fitness facility. A limitation was that the checklist tool could not be utilised until week 12; earlier observation could have provided more timely formative feedback and is recommended in future studies. It was also evident from the qualitative observations that the program was well regarded by participants and that participant safety was ensured in using gym equipment and in a culturally safe environment. A higher number of program checklist items were achieved in the first cohort and there were some qualitative differences such as cohort one having a greater focus on strength and conditioning activities whereas cohort two included other activities like basketball that may be due to the different staff qualifications and experience. Staff member characteristics and qualifications data indicated higher resourcing in terms of the number of overall program staff, Aboriginal staff and both strength and conditioning and nutrition qualified staff in the first cohort than the second cohort that impacted the safety, cultural relevance of the program implementation. Having program staff support, respect, encouragement and positive role models are known interpersonal, relational facilitators of physical activity participation [23]. It is recommended that future program implementation be resourced consistent with the program co‐design and ensure Aboriginal leadership in all program aspects, guidance that previous literature supported [12, 24].

However, the PCYC data for the 9 months after program delivery, collected for cohort one only, indicates that gym attendance was low at post implementation follow‐up. This finding underscores the importance of a structured, instructor led group‐based program for young people who may require the program model for a longer period to develop sustainable participation practices, consistent with other programs' sustainability challenges [24]. Cohort 1 identified and fostered participant leadership, which translated to some participants being mini‐group leads and role modelling. Some of these participants returned to Cohort 2 as experienced leaders, supporting staff and sustainability. Therefore, it is recommended that programs empower participants by identifying natural leaders and providing mentorship opportunities, including leadership training and capacity building sessions within the program and should include offering pathways to strength and conditioning or fitness qualifications for participants. This is consistent with another physical activity program, where mentoring and capacity building has been integral to a program for young adults, the Indigenous Marathon Program [25] that has contributed to local Aboriginal and Torres Strait Islander led community program sustainability [26].

Qualitative yarning findings found that the program provided a quality, suitable facility and was successful in addressing barriers such as transport and attendance costs that have been identified in previous reviews of physical activity studies with Aboriginal and Torres Strait Islander children [27] and adults [23]. The 14‐week period, was effective in improving fitness and wellbeing outcomes [16] and was sufficient for participants to develop the comprehensive knowledge, skills and confidence necessary to continue strength and conditioning activities independently. However, the post‐program paid membership removed cost as a barrier during the program and up to 6‐months post, but once the subsidised membership ceased, ongoing attendance significantly reduced. Further, the lack of the PCYC Community Bus for transport to attend the facility post‐program likely contributed to lower post‐program attendance, underscoring the need for ongoing transport provision for young people to attend sporting facilities that has been described previously [28]. The school and community partnerships within the Young Strong and Deadly program likely contributed to the successful recruitment and engagement so ongoing collaboration with schools and local organisations are recommended, consistent with previous literature [28], to reduce external constraints.

The different methods used within this process evaluation led to achievement of different components of the program logic model beyond the pre‐post impact evaluation [16]. For example, the objectively measured improvements in strength and conditioning outcomes were supported by participant, family and stakeholder views evident in the qualitative yarning data, where participants described feeling stronger and fitter. The quantitative survey findings also found improved wellbeing [16] that were also supported by the qualitative yarning data of improved confidence that has been previously demonstrated in the limited existing evidence base [11].

Some benefits of the nutrition program components were also described in the qualitative yarning results. Enhancing the program with more comprehensive health education, such as nutrition workshops, would provide participants with essential knowledge to support their overall wellbeing and holistic program offerings have been demonstrated in another recent local study [29]. By offering culturally relevant examples and addressing barriers to healthy eating within Aboriginal communities, particularly in urban environments, the program can empower participants to make informed choices and healthy lifestyle improvements alongside their physical training.

A limitation of the data informing the nutrition findings was that researcher observation of the nutrition sessions that took place in specific weeks at an alternative program location was not logistically feasible to undertake. The evaluation was also limited by the amount and depth of data collected. Qualitative responses were often brief, consistent with the communication style of the teenage participants. Findings relating to nutrition and SEWB were primarily based on qualitative data; therefore, reported changes should be interpreted as perceived rather than definitive outcomes. Despite these limitations, the evaluation provides useful practical insights to inform the delivery of similar programs with young people in community settings.

## 6 Conclusion

Young Strong and Deadly has demonstrated feasibility and acceptability among Aboriginal young people and their families. This process evaluation demonstrated achievement of most program logic model activities, outputs and short‐term outcomes including teaching young people how to perform physical activity safely and effectively in a gym environment, healthy food education and meal preparation activities, improved fitness and wellbeing, health seeking behaviour and engagement with community‐based health services. Ongoing program implementation and evaluation, drawing on the learnings from this process evaluation, will increase the likelihood of achieving the long‐term outcomes in reducing chronic disease and associated risks through a culturally responsive program, giving lasting benefits to the next generation of young Aboriginal and Torres Strait Islander people.

## Funding

This work was supported by Western Sydney Local Health District.

## Ethics Statement

Ethics approval was obtained from the Sydney Children's Hospitals Network (SCHN) Human Research Ethics Committee (2023/ETH00583) and the Aboriginal Health & Medical Research Council Human Research Ethics Committee (2124/23).

## Consent

Informed consent was obtained from all participants.

## Conflicts of Interest

The authors declare no conflicts of interest.

## Data Availability

The data that support the findings of this study are available on request from the corresponding author. The data are not publicly available due to privacy or ethical restrictions.

## Appendix 1 Young Strong and Deadly Program Logic

**Table:**

Inputs	Activities	Outputs	Impact—short term outcomes	Long term outcomes
Exercise Program; Personal trainers for 4 h per week Weekly Gym membership at Police and Community Youth Club—Shalvey Campus, NSW Subscription for 6–9 months Promotional collateral (flyers, uniforms etc.) PCYC bus transportation for young people	Implement a novel pilot adolescent exercise and education program Deliver a culturally appropriate, guided physical activity program for young people for 14 weeks Provide participants with a gym membership for the period of the program (14 weeks) and ongoing for the period of follow up (3‐6 months) Provide transportation using the PCYC bus to transport young people to the gym from a central location around Blacktown/Mount Druitt LGA Assess and monitor participant wellbeing, physical activity and nutrition behaviour and provide support and referral to appropriate services, if needed Teach young people how to perform physical activity safely and effectively in a gym environment	Ongoing gym membership Ongoing participation in physical exercise Documentation of wellbeing, physical activity and nutrition behaviour in electronic medical records Electronic record of youth participation in program Safe gym practices by participants	Young Aboriginal people are knowledgeable of physical exercise protocols Young Aboriginal people routinely engage in physical activities Improved understanding of barriers and facilitating factors for Aboriginal young people to participate in health promoting activities Improved cardiorespiratory fitness, control weight Improved feelings of personal wellbeing in Aboriginal young people	Reduced chronic disease risk factors in young Aboriginal people A sustainable physical activity and nutrition education program is implemented by WSLHD for young people including culturally appropriate services for Aboriginal young people Improved health seeking behaviour among Aboriginal young people
Measurement tools—Food Frequency Questionnaires and Physical activity measurements The Heart Foundation's Koori Cooking trailer, cooking equipment and toolkit Nutritionist facilitated food and nutrition literacy education content and delivery	Host four (4) dedicated health and nutrition education sessions Sessions contain education on food and nutrition literacy: Nutrition literacy Basic nutrition concepts (macronutrients, key micronutrients) Portion size guidance and Australian food group serves recommendations Reading and interpreting food labels and ingredient lists Understanding how food choices affect growth, strength and health Identifying credible nutrition information and marketing claims Food literacy Meal planning and food budgeting skills Selecting foods within available food environments Cooking techniques and recipe skills Safe food handling and storage Participants undertake guided preparation and cooking of healthy meals from the Koori Cook‐Off toolkit Invite Family members to attend and participate in sessions on how to cook healthy meals Provide completed meals to participants and their families	Healthy meals training attended by program participants (and their family members) Healthy meals prepared Program participants knowledgeable and consume health meal choices Health service attendance by participants for further support regarding their health	Improved understanding of barriers and facilitating factors for Aboriginal young people to eat nutritious foods Improve food literacy among Aboriginal young people Improved intake of nutritious food among Aboriginal young people Improve health seeking behaviour and engagement with community‐based health services among Aboriginal young people	
Trainer, paint and t‐shirts	Cultural Painting Workshop—engaging youth in painting t‐shirts and creating non‐specific promotional material	Paintings non‐specific promotional material	Non‐specific promotional material created	

*Note:* For the purposes of this paper, family is used as an inclusive term reflecting Aboriginal and Torres Strait Islander kinship systems, which encompass extended networks of significant people who may or may not be biologically related.

Abbreviations: PCYC: Police and Community Youth Club; WSLHD: Western Sydney Local Health District.

## Appendix 2 Yarning Questions at End of Program and 3 Month Follow‐Up

**Table:**

Program participants: What will make you want to or what made you to continue? What may stop you from or interrupt you continuing? Would you recommend the program to a friend? How likely is it that you will continue to participate in physical activity programs/healthy eating? What would make this easier? Do you have any other suggestions for improvement? Family and Stakeholders: What was your experience of the program, in your role? What has been the impact of the program on participants? Have there been reasons that make it hard for participants to come to the program? Have there been any challenges in implementing the program? What has been the impact of the program (on your organisation)? Has the program been appropriate for participants in terms of culture, age and any other factors? Would you recommend the program to other organisations/communities? Why/why not?
